# The structural and optical properties of type III human collagen biosynthetic corneal substitutes

**DOI:** 10.1016/j.actbio.2015.07.009

**Published:** 2015-10-01

**Authors:** Sally Hayes, Phillip Lewis, M. Mirazul Islam, James Doutch, Thomas Sorensen, Tomas White, May Griffith, Keith M. Meek

**Affiliations:** a Structural Biophysics Group, Cardiff Centre for Vision Science, Cardiff University, Wales CF24 4HQ, UK; b Cardiff Institute for Tissue Engineering and Repair (CITER), Cardiff University, Wales CF24 4HQ, UK; c Swedish Medical Nanoscience Center, Dept. of Neurosciences, Karolinska Institutet, S-17177 Stockholm, Sweden; d Integrative Regenerative Medicine Centre, Dept. of Clinical and Experimental Medicine, Cell Biology Bldg. – Level 10, Linköping University, S-58185 Linköping, Sweden; e Diamond Light Source, Didcot, Oxfordshire OX11 ODE, UK

**Keywords:** Cornea, Collagen, Transplantation, Recombinant protein

## Abstract

The structural and optical properties of clinically biocompatible, cell-free hydrogels comprised of synthetically cross-linked and moulded recombinant human collagen type III (RHCIII) with and without the incorporation of 2-methacryloyloxyethyl phosphorylcholine (MPC) were assessed using transmission electron microscopy (TEM), X-ray scattering, spectroscopy and refractometry. These findings were examined alongside similarly obtained data from 21 human donor corneas. TEM demonstrated the presence of loosely bundled aggregates of fine collagen filaments within both RHCIII and RHCIII-MPC implants, which X-ray scattering showed to lack D-banding and be preferentially aligned in a uniaxial orientation throughout. This arrangement differs from the predominantly biaxial alignment of collagen fibrils that exists in the human cornea. By virtue of their high water content (90%), very fine collagen filaments (2–9 nm) and lack of cells, the collagen hydrogels were found to transmit almost all incident light in the visible spectrum. They also transmitted a large proportion of UV light compared to the cornea which acts as an effective UV filter. Patients implanted with these hydrogels should be cautious about UV exposure prior to regrowth of the epithelium and in-growth of corneal cells into the implants.

## Graphical abstract

**Figure:**
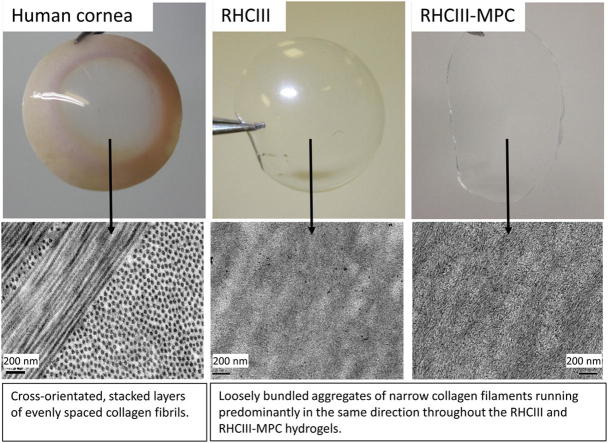

## 1 Introduction

The intrinsic properties of the healthy cornea, namely its strength, transparency and precise curvature enable it to withstand external damage and resist intraocular pressure, whilst also allowing it transmit over 95% of incoming light [1] and provide 70% of the focussing power of the eye. These properties are largely governed by the unique architecture of the corneal stroma, which measures approximately 500 μm in thickness and occupies 90% of the total corneal thickness. The physiologically hydrated stroma consists primarily of water (78%) and the protein collagen (15%) [2]. Collagen in the corneal stroma is predominantly type I, with smaller amounts of types V, VI, XII, XIII, XIV and XXIV; the existence of type III collagen in the healthy, unwounded corneal stroma is still debated [3]. Corneal transparency is dependent on the specific arrangement of collagen within the stroma, whereby narrow (∼32 nm [4]), evenly spaced collagen fibrils lie parallel to each other in cross-orientated stacked layers (lamellae). One of the main ultra-structural features of ‘native’ collagen fibrils is the presence of alternating gap and overlap regions along the fibril (D-periodicity), arising from the staggered association of adjacent collagen molecules [5]. This D-periodic arrangement is critical for the formation of heterotypic structures consisting of fibrillar collagens and non-collagenous macromolecules. Proteoglycans bind at specific sites along the fibrils [6,7] and play a role in fibril assembly, matrix organisation and ultimately corneal transparency [8]. Small-angle X-ray scattering patterns generated from corneal collagen produce a series of so-called “meridional” X-ray reflections coming from the D-periodicity and indexing on about 65 nm [9]. The cornea, unlike most connective tissues, also produces a small-angle “equatorial” pattern which is caused by the uniformity of fibril diameters and the regular spacing of collagen fibrils (over a short range), within each lamella of the corneal stroma [10]. Further information about collagen organisation within the cornea can be ascertained from wide angle equatorial X-ray scattering, which arises from the lateral packing of the molecules within the collagen fibrils. Using this technique it has been shown that human corneal collagen has an average Bragg intermolecular spacing of 15 Å [11] and that lamellae in the central region of the human cornea (in the deeper stromal layers [12]), are predominantly aligned in the superior-inferior and nasal-temporal directions [13–15]. It is thought that this arrangement, which is not seen in lower visual acuity species [16], may be necessary to resist the forces of the four major extraocular muscles which insert into the sclera at opposing positions along each meridian.

A loss of vision due to corneal trauma or disease affects over 19 million individuals worldwide. For many, transplantation is the only treatment, but the supply of quality donor tissue cannot meet demand. This has prompted an intense effort to develop alternatives, the most promising of which are collagen-based hydrogels [17–20]. Laboratory based studies comparing the efficacy of recombinant human collagen types I and III as corneal substitutes for implantation, showed that the chemical and mechanical properties of the hydrogels was similar; however, the implants manufactured from synthetically cross-linked recombinant human collagen type III (RHC-III) were found to possess superior optical clarity [21]. Subsequent in vivo studies, using cell-free RHCIII moulded implants, have shown the implants to remain stable and avascular for over 48 months following implantation into 10 human subjects as a partial thickness graft. The implants served as scaffolds, allowing endogenous cells from the patient to enter and populate it to form neo-corneal tissue comprising an epithelium, stroma and nerves in all cases [22,23]. Further development of the biosynthetic corneal hydrogel, with the incorporation of 2-methacryloyloxyethyl phosphorylcholine (MPC), a synthetic phosphorylcholine polymer, to form an interpenetrating collagen-phospholipid network (RHCIII-MPC), has led to enhanced stability, particularly enzymatic resistance, although the tensile strength of the construct remains considerably inferior to that of the human cornea [24].

Although the triple helical recombinant collagens used to create the hydrogels are known to contain the same amino acid sequences and an equivalent extent of proline hydroxylation as naturally occurring human collagens, the molecular and fibrillar arrangement of collagen within the manufactured hydrogels has yet to be determined. Here, wide- and small-angle X-ray scattering, transmission electron microscopy (TEM), spectroscopy and refractometry have been used to determine the ultrastructure, refractive index and light transmission properties of crosslinked RHCIII hydrogels (with and without MPC). The findings are examined alongside new and previously published data from human corneas.

## 2 Material and methods

### 2.1 Production of biosynthetic hydrogels

RHCIII hydrogels were prepared by crosslinking with N-(3-dimethylaminopropyl)-N′-ethylcarbodiimide as previously described in detail [25] but with a higher starting concentration of 18% (wt/wt). Briefly, 0.5 ml (∼500 mg) solution of 18% (wt/wt) RHCIII (FibroGen Inc., San Francisco, CA) was buffered with 150 μl of 0.625 M MES in the syringe mixing system. EDC and NHS was then added to crosslink the collagen. Molar equivalent ratio of collagen-NH_2_:EDC was 1:0.4 and EDC:NHS was 1:1. NHS and EDC solutions were made in MES buffer in concentration of 10% (wt/vol) and 5% (wt/vol), respectively.

The RHCIII-MPC interpenetrating polymer network was fabricated as previously described [26]. Briefly, a 450–500 mg of 18% (wt/wt) aqueous solution of sterile recombinant human collagen type III (RHCIII; FibroGen Inc., San Francisco, CA) was thoroughly mixed with 150 μl of 0.625 M 2-morpholinoethane sulphonic acid monohydrate (MES) buffer within a syringe mixing system and kept cool on ice (0 °C). A 200 μl solution of 2-methacryloyloxyethyl phosphorylcholine (MPC, Paramount Fine Chemicals Co., Ltd., Dalian, China) in 0.625 M MES (RHCIII:MPC, 2:1 (wt/wt)), followed by poly(ethylene glycol) diacrylate (PEGDA:MPC (wt/wt) = 1:3) (PEGDA Mn.575; Sigma–Aldrich, MO) were added and mixed within the syringe mixing system to form a second polymeric network. Calculated volumes of 4% (wt/vol) ammonium persulphate (APS; Sigma) and 2% (wt/vol) N,N,N,N-tetramethylethylenediamine (TEMED; Sigma) solution in MES were added sequentially; (APS/MPC (wt/wt) = 0.03:1, APS:TEMED (wt/wt) = 1:0.77). After mixing thoroughly, calculated amounts of MES buffered N-(3-dimethylaminopropyl)-N′-ethylcarbodiimide (EDC; Sigma–Aldrich, MO) and N-hydroxysuccinimide (NHS; Sigma–Aldrich, MO) were added. The proportions of reactants were as follows – 5% EDC (wt/vol) and 10% NHS. The ratio EDC:RHCIII-NH_2_ used was 0.4:1 (mol:mol), while ratio of EDC:NHS (mol:mol) = 1:1. The solution was mixed thoroughly at 0 °C.

**Fig. 1:**
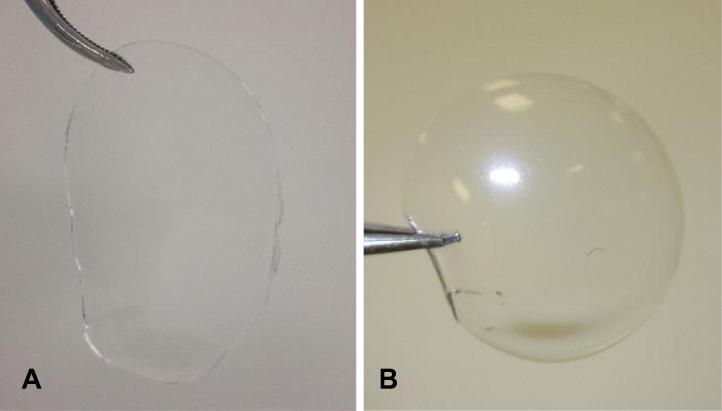
The light transmission properties and the structural characteristics of the hydrogels were characterised using sheets (A) and moulded cornea-shaped implants (B).

In both RHCIII and RHCIII-MPC, the mixed solution were cast into flat sheets (Fig. 1A) or cornea-shaped implants (500 μm thick, 12 mm diameter) (Fig. 1B) by dispensing into flat glass moulds or curved polypropylene moulds, respectively. Both were cured at 100% humidity at 21 °C for up to 24 h and then at 37 °C for up to 24 h. After demoulding, the hydrogels were washed thoroughly in 0.1 M Na_2_HPO_4_ for 3 to 4 h and then in 0.1 M PBS for up to one week to remove all unreacted chemicals. The hydrogels were then stored in PBS containing 1% chloroform to maintain sterility prior to use.

### 2.2 Human corneas

This project was approved by the NHS Research Ethics Committee in January 2012 and was carried out in accordance with the tenets of the Declaration of Helsinki.

One ocular globe from a 69 year old female (A1), was obtained from the UK Transplant Service, Bristol Eye Bank. The eye was enucleated within 20 h of death but declared unsuitable for transplantation. Twenty hours later the globe was submerged in 4% paraformaldehyde. After fixation the cornea was dissected and processed for electron microscopy.

A further seventeen human donor corneas with an intact scleral rim (B1–14; C1–3) were also obtained from the Bristol Eye Bank. These corneas were provided for research after being deemed unsuitable for transplantation due to low endothelial cell count following one month of storage in culture medium. An 8 mm disc was trephined from the centre of eleven of the corneas (B1–11, aged 64–84 years). The corneal discs were then individually placed into 14KD cut-off dialysis tubing and immersed in varying concentrations of polyethylene glycol solution (0–30%) containing 0.15 M NaCl. The corneal discs were allowed to equilibrate for 3 days at 4 °C. This equilibration technique is an effective method of accurately adjusting the hydration of corneal tissue without any loss of proteoglycans and is therefore considered the method of choice for examining the relationship between corneal ultrastructure and hydration [27]. The equilibrated corneas were used for wide-angle X-ray scattering studies examining the effect of tissue hydration on collagen intermolecular spacing.

Three cornea-scleral discs were used for wide-angle X-ray scattering to examine changes in collagen orientation across the tissue (B12–B14, aged 76–84 years). Corneas with intact scleral rims were used for this part of the study to avoid the complication of edge-effect changes in collagen orientation that can occur during the trephination of corneal buttons (unpublished data). Although we have previously shown that corneal swelling does not alter the predominant orientation of collagen within the cornea, it does have the effect of reducing X-ray scatter intensity [28], and for this reason it was necessary to counteract the corneal swelling that occurred as a result of culture medium storage. As the dialysis equilibration technique described previously (to adjust the hydration of trephined corneal discs) cannot be used effectively to adjust the hydration of cornea-scleral discs (due to their curvature), tissue hydration was reduced by adding 5% dextran to the medium two days prior to X-ray data collection.

Small-angle X-ray scattering studies were performed on 8 mm discs trephined from the centre of three corneas (C1–3, aged 52, 74 and 78 years). As small-angle X-ray scattering is particularly sensitive to changes in corneal hydration it was necessary to examine the corneal discs at close to physiological hydration; this was achieved using a 1–3% polyethylene glycol solution and the equilibration technique described previously for samples B1–11.

### 2.3 Characterisation of optical properties

The refractive indices of three RHCIII-MPC hydrogel sheets and their PBS storage medium were measured at 19 °C and a wavelength of 589 nm, using a bench-top Abbe 60 series Refractometer (Bellingham and Stanley, London, UK) which was calibrated against a silica test plate of known refractive index.

During spectrophotometry measurements it was necessary to immerse the hydrogel sheets in a bathing medium of similar refractive index to maintain their hydration whilst also minimising the potential for surface reflections. As the PBS storage medium was found to have a similar refractive index to that of the hydrogels, it was deemed to be a suitable bathing medium for spectrophotometry measurements.

A 5 × 15 mm strip of the hydrogel sheet was placed in a quartz cuvette (filled with the PBS storage medium) and positioned in the spectrophotometer (U-2800 UV–VIS spectrophotometer with Hitachi UV Solutions software, Hitachi High Technologies, Berkshire, UK), such that the beam was perpendicular to the hydrogel. Using two spectral lamps (deuterium lamp for 230–315 nm and a tungsten-halogen lamp for 315–1110 nm), continuous light absorption measurements were made between 1100 and 230 nm for each of the three RHCIII-MPC hydrogel sheets. A baseline reading from the cuvette containing only the storage medium was deducted from each hydrogel measurement. Absorption readings were then converted to percent light transmission for display purposes.

The light transmission data obtained from the RHCIII-MPC hydrogels were examined alongside similar previously published data from human corneas and RHCIII hydrogels.

### 2.4 Characterisation of structural properties

#### 2.4.1 Standard and high pressure freezing TEM

Cornea A1 and one half of a RHCIII and a RHCIII-MPC hydrogel underwent standard processing for TEM, whereby the specimen was dehydrated through an ascending ethanol series before being embedded in Araldite resin and polymerised at 60 °C for 24 h. The other half of the RHCIII and RHCIII-MPC hydrogels were processed for TEM using a high pressure freezing technique specifically designed to avoid preparation artefacts that may occur during standard TEM processing [29]. These samples were trimmed to 1.2 mm and rapidly cryofixed in a Leica EMPACT2 high pressure freezer. Frozen specimens were freeze substituted at −80 °C in 2% Osmium/Acetone for 24 h, and embedded in Araldite resin at 20 °C. From each embedded/frozen specimen, ultrathin sections (∼110 nm thick) were cut with a diamond knife on a Leica Ultracut UC6 ultramicrotome. Sections were collected on peloform-coated slot grids, stained with saturated aqueous uranyl acetate for 30 min at room temperature, Lead citrate for 7 min, and examined at 80 kV in a JEOL 1010 transmission electron microscope (Japan) equipped with a bottom-mount 14-bit CCD camera (Orius SC1000 Gatan, Pleasanton, CA).

#### 2.4.2 Wide-angle X-ray scattering

On beam line I02 at Diamond Light Source (Oxfordshire, UK), a 0.5–1 s exposure to an X-ray beam measuring 80 μm vertically by 100 μm horizontally and with a wavelength of 0.1 nm was used to collect a single wide-angle X-ray scatter pattern from the centre of each of the eleven human corneas (B1–11) that had been equilibrated to a tissue water content of between 37% and 68%. Using the same experimental set-up single X-ray scatter patterns were also obtained from the centre of three RHCIII and three RHCIII-MPC moulded hydrogels, in their naturally hydrated state (89–91% water) and at various stages of dehydration (down to ∼35%). Once a collagen inter-molecular reflection was obtained from the centre of each hydrogel (usually when the water content was below 72%), further X-ray scatter patterns (∼250 images) were collected at 0.5 mm intervals in a grid pattern across the entire specimen. In a similar fashion, wide-angle X-ray scattering data was collected at 0.5 mm intervals in an 11 × 11 mm grid pattern across two human corneas with intact scleral rims (B12–13). All X-ray scatter patterns were recorded on a Pilatus detector positioned 28 cm behind the specimen.

Wide-angle X-ray scattering data was also obtained at 0.5 mm intervals across the central 8 mm of a single human cornea (B14) on Station 14.1 at the former UK Synchrotron Radiation Source (Daresbury, UK). In this case, each X-ray scatter pattern was generated using a 45 s exposure to a 0.1488-nm wavelength X-ray beam measuring 200 × 200 μm, and recorded on a charge-coupled (CCD) X-ray detector (ADSC, Poway, CA) placed 15 cm behind the specimen.

In order to prevent tissue dehydration during X-ray data collection each sample was wrapped tightly in polyvinylidene chloride catering film and placed into sealed sample holder enclosed between two sheets of Mylar (DuPont Teijin Films™, London, UK). The percentage water content of each specimen at the time of data collection was calculated using measured wet and dry weights (recorded immediately prior to sample mounting and after 5 days of drying at 60°).

The angular distribution of scatter intensity around the collagen intermolecular reflection was measured to form a 0–360° distribution pattern using MATLAB software (Mathsworks, UK). The distance from the centre of the scattering pattern to the intermolecular reflection was measured and calibrated against the 0.304 nm reflection of powdered calcite in order to obtain the Bragg intermolecular spacing of collagen.

The total area under the intensity profile (i.e. the total X-ray scatter) is proportional to the total amount of fibrillar collagen in the path of the beam and is comprised of isotropic scatter and non-isotropic (preferentially aligned) scatter. In the cornea, most collagen lamellae lie more or less parallel to the surface of the tissue and the isotropic scatter arises from collagen fibrils that lie equally in all directions within the plane of the tissue. The same is also true for the RHCIII and RHCIII-MPC constructs in which SEM observations have shown a lamellar arrangement to exist [26].

In both the cornea and the hydrogels, the preferentially aligned scatter arises predominantly from collagen within the sample plane that adopts a preferred orientation. In order to show the predominant orientation of collagen within each cornea and hydrogel, the contribution of isotropic scatter was removed from the total scatter intensity profile to leave only the scatter from preferentially aligned collagen. The remaining profile of aligned collagen scatter was then shifted by 90° (to account for the fact that, in the equatorial pattern, X-rays are scattered at right angles to the orientation of the collagen molecules) and subsequently displayed as a vector plot in which the distance from the origin to the edge, at any given angle, represents the intensity of scatter from preferentially aligned collagen lying in a particular direction. Vector plot maps showing the preferred orientation of aligned collagen throughout each sample were formed by compiling individual vector plots onto a grid relating to their position within the construct. For further details on this wide-angle X-ray scattering data analysis technique the reader is directed to a review by Meek and Boote [30].

The percentage of the total X-ray scatter arising from preferentially aligned collagen was calculated by first summing the area under each intensity profile to produce a numeric value of scatter intensity for both total collagen and aligned collagen and then entering the values into the equation: (aligned intensity/total intensity) * 100. In this way, a value of 100% would indicate that all collagen is preferentially aligned within the plane of the sample.

#### 2.4.3 Small-angle X-ray scattering

On beam line I22 (Diamond Light Source, UK), small-angle X-ray scatter patterns were recorded from the centre of three human corneas (C1–3) and three RHCIII moulded hydrogels. Unfortunately, small-angle X-ray scattering data could not be collected from the RHCIII-MPC hydrogels due to a lack of specimen availability at the time of our scheduled access to the beam line.

X-ray scattering data was collected from the human corneas and the RHCIII hydrogels in their natural hydration state using a 15 s exposure to an X-ray beam measuring 200 μm by 200 μm with a wavelength of 1.1 Å. In order to enhance visualisation of any collagen D-periodicity, the hydrogels were then soaked in 2% phosphotungstic acid (PH 1.7) for 20 min, followed by a 5 min dH_2_O wash. Further X-ray scatter patterns were generated at 1 mm intervals in a 3 × 3 mm grid pattern across the centre of each specimen. All X-ray patterns were recorded on a detector positioned 6.2 m behind the sample.

The small-angle X-ray scatter patterns were analysed as described previously [4], using a graphics package (MATLAB, MathsWorks, UK). Each X-ray scatter pattern was circularly integrated to generate an X-ray scatter intensity profile, from which background scatter from non-fibrillar tissue components was removed by fitting and then subtracting a square-power law background curve. The radial position of the diffuse equatorial scatter (evident in the phosphotungstic acid stained specimens only) was calibrated against the 67 nm D-periodicity of collagen in hydrated rat tail tendon to produce measurements of average fibril diameter across the construct.

### 2.5 Determination of corneal and hydrogel hydration

The wet weight of the corneal buttons (B1–11; C1–3), RHCIII and RHCIII-MPC hydrogels were recorded before and after data collection, allowing an average wet weight during data collection to be calculated. Following completion of each study, the samples were dried in a 60 °C oven for 7 days until a constant dry weight was achieved. The percentage water content of each specimen was calculated using the equation: ((average wet weight-dry weight)/average wet weight) × 100.

### 2.6 Statistical analysis

All statistical data are expressed as a mean ± standard deviation. Data were analysed using a one-way ANOVA followed by post hoc Tukey’s multiple comparison tests. For all comparisons, *p* < 0.05 was considered statistically significant.

## 3 Results

### 3.1 Refractive index

All RHCIII-MPC hydrogel sheets were between 88% and 90% hydrated at the time of examination and had an average refractive index of 1.334 ± 0.0003. Using published refractive index measurements for human corneal tear film [31], epithelium and stroma [32], the Fresnel reflection at an incidence angle normal to the corneal surface was theoretically calculated for each of the boundaries in a normal healthy cornea and a RHCIII-MPC hydrogel implanted cornea (following epithelial regrowth) (Table 1). The total percent of the incident light reflected from these boundaries was calculated to be 2.06% in a healthy human cornea and 2.14% in an RHCIII-MPC implanted cornea. Based on a previously published value of 1.35 for the refractive index of RHCIII hydrogels [21], the total percent of incident light reflected from these boundaries in a RHCIII implanted cornea was calculated to be 2.09%.

As the PBS storage medium surrounding the hydrogels was found to have a refractive index (1.335) similar to that of the hydrogels (1.334), it was deemed suitable for use as a hydrating medium in light transmission studies.

### 3.2 Light transmission

**Fig. 2:**
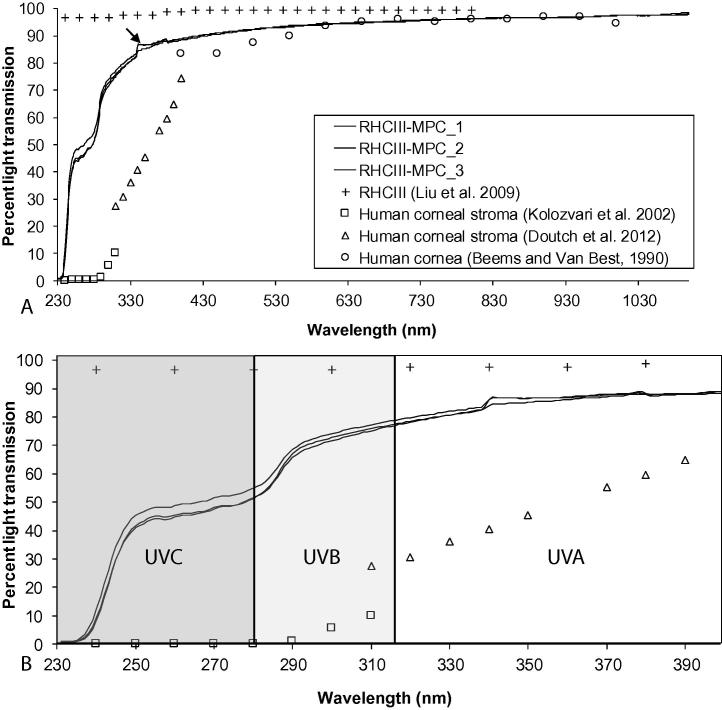
Light transmission spectra from three RHCIII-MPC hydrogels shown alongside published data from RHCIII hydrogels (Liu et al. [24]) and human cornea (Beems and Van Best [1]) and stroma (Kolozvari et al. [35] and Doutch et al. [34]). The light transmission spectra of the hydrogels was measured continuously between 230 and 1030 nm (A). A large difference in light transmission was observed between the hydrogels and the human cornea in the UV light region between 230 and 400 nm (B). The small anomaly in the spectra at 340 nm (arrow) is due to the change of light source from a tungsten-halogen lamp to a deuterium lamp.

There was no significant difference in the light transmission spectra obtained from each of the three RHCIII-MPC hydrogels examined (Fig. 2A). In the visible spectrum (between 400 and 700 nm) each RHCIII-MPC hydrogel transmitted between 89% and 95% of incident light, and in the middle of the spectrum (at 500 nm) 92 ± 0.1% of light was transmitted (Fig. 2A). These values are similar to those previously reported for human corneas [1] and RHCIII hydrogels [33]. Interestingly, the RHCIII-MPC hydrogels were also found to transmit a large proportion of UV light (Fig. 2B). In particular, the RHCIII-MPC hydrogels transmitted on average 85 ± 3% of UVA, 70 ± 7% of UVB light and 48 ± 3% of UVC between 250 and 280 nm. Although the UV transmittance of the RHCIII-MPC hydrogels was found to be substantially less than that of the RHCIII hydrogels (which transmit ∼97% of UVA, UVB and UVC) [33], the optical properties of the both hydrogels over this wavelength range remain in stark contrast to the human cornea, which shows reduced light transmission when the wavelength decreases from 400 to 330 nm (UVA) [34] and transmits very little UVB (∼6%) and no UVC (Fig. 2B) [35].

### 3.3 Structural properties

#### 3.3.1 Transmission electron microscopy

**Fig. 3:**
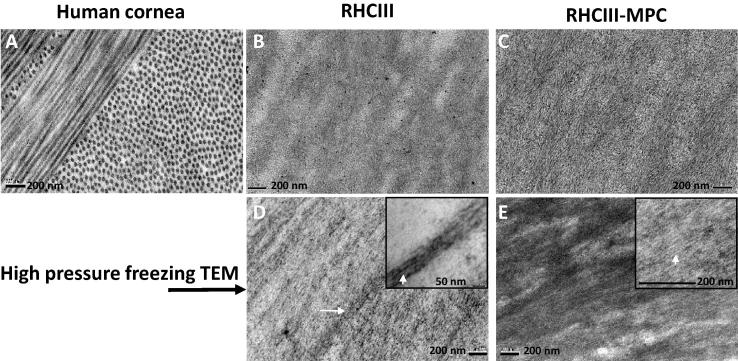
Conventional TEM images show that human corneal collagen is regularly spaced and arranged in cross-orientated layers (A). Standard TEM (B and C) and high pressure freezing TEM (D and E) demonstrate the presence of aggregates of loosely bundled smaller collagen filaments (highlighted by white arrows in D and E) that are aligned in a predominantly uniaxial orientation throughout the RHCIII and RHCIII-MPC hydrogels.

The human cornea contains cross-orientated, stacked layers of evenly spaced collagen fibrils (Fig. 3A). Standard processing TEM images of the RHCIII and RHCIII-MPC hydrogels revealed the presence of loosely bundled aggregates of narrow collagen filaments (Fig. 3B and C). TEM images of the RHCIII and RHCIII-MPC hydrogels taken after high pressure freezing (Fig. 3D and E) showed less collagen aggregation than seen in the standard processing TEM images (Fig. 3B and C). The non-clumped filaments in the high pressure frozen RHCIII and RHCIII-MPC hydrogels had a diameter of between 2 and 9 nm and the clumped aggregates were found to measure between 40 and 60 nm in diameter. A strong alignment of collagen filaments was observed in all TEM images of the hydrogels (Fig. 3B–D), irrespective of the processing technique employed, thereby confirming that the alignment of collagen filaments is a real property of the material and not a processing artefact. Detailed examination of electron micrographs taken from the hydrogels at high magnification (50,000×) revealed no evidence of the collagen D-banding that is characteristic of native fibrillar collagen.

#### 3.3.2 Wide-angle X-ray scattering

**Fig. 4:**
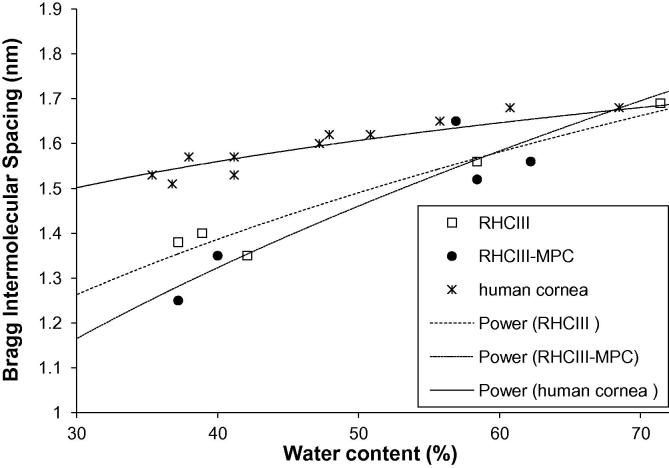
The relationship between hydration and collagen intermolecular spacing in human corneas (B1–B11) and RHCIII hydrogels (with and without MPC). A power-law trend line has been fitted to each data set.

Wide-angle X-ray scatter patterns, arising from the packing of collagen molecules within the fibres of the hydrogels, were only obtainable when the hydrogel was dehydrated to a water content of below 72%. As is known to be the case in bovine corneas [27], and here shown in human corneas, a reduction in hydration led to a decrease in the average Bragg intermolecular spacing of collagen (Fig. 4). This was also found to be the case with the hydrogels, however, the precise relationship between collagen intermolecular spacing and tissue hydration differed between the corneas and the hydrogels (Fig. 4). The relationship between tissue hydration and collagen intermolecular spacing did not appear to be affected by the presence or absence of MPC within the hydrogel (Fig. 4).

**Fig. 5:**
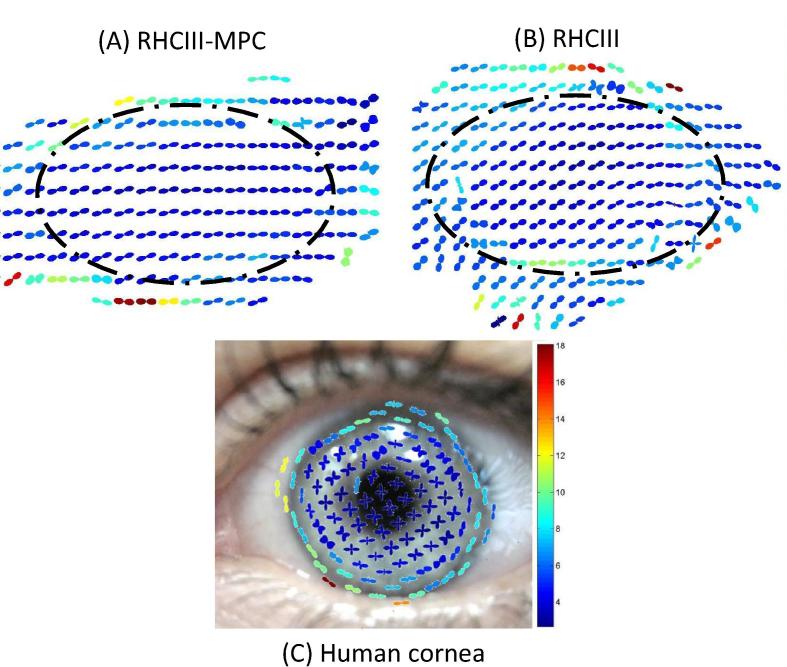
Vector plot maps showing the predominant orientation of collagen at 0.5 mm intervals throughout RHCIII corneal hydrogels (with (A) and without (B) MPC). Changes in collagen orientation outside the broken line are drying artefacts. For comparative purposes, a vector plot map of preferential collagen alignment at 1 mm intervals across a human cornea (specimen B12) is shown superimposed onto a picture of the eye (C). The colour scale is used to depict the relative amount of preferentially aligned collagen at a particular position within an individual specimen (red vector plots indicate the greatest amount of alignment).

With the exception of a few drying artefacts at the edges of the hydrogels, collagen was found to be preferentially aligned in a uniaxial orientation throughout both the RHCIII-MPC (Fig. 5A) and RHCIII hydrogels (Fig. 5B). This collagen arrangement differs from the predominantly biaxial alignment of collagen known (and shown here) to exist in the central human cornea (Fig. 5C). Preferentially aligned collagen was found on average to account for 45 ± 5% and 51 ± 9% of the total X-ray scatter intensity in the centre of the RHCIII and RHCIII-MPC hydrogels respectively and 29 ± 1% in the centre of the human cornea (based on samples B12–B14). Statistical analysis revealed the proportion of preferentially aligned collagen to not differ significantly between RHCIII and RHCIII-MPC hydrogels but to be significantly higher in the hydrogels than the human cornea (*p* < 0.05).

#### 3.3.3 Small-angle X-ray scattering

**Fig. 6:**
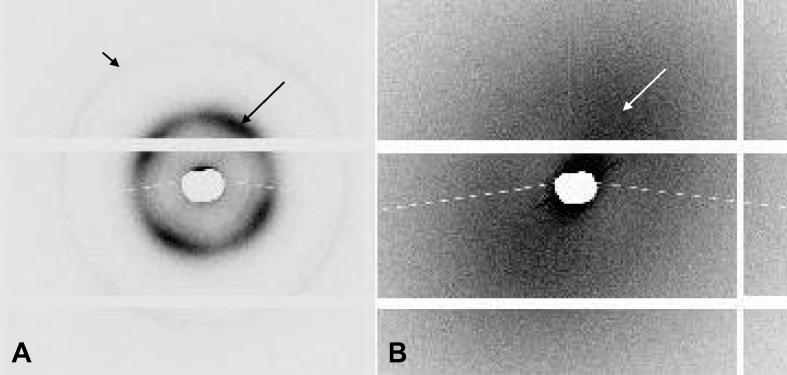
Small-angle X-ray scatter pattern obtained from the centre of a human cornea (A) exhibits sharp meridional reflections arising from the regular D-period along the collagen fibril axis (3rd order reflection is highlighted by a short black arrow). The human cornea also produces a four lobed equatorial reflection (long black arrow) arising from the regular spacing of uniformly narrow collagen fibrils which are preferentially aligned biaxially within the stroma. X-ray scatter patterns from similarly hydrated, negatively stained RHCIII biosynthetic hydrogels (B) showed diffuse equatorial scatter (highlighted by a white arrow) with two lobes of increased X-ray scatter, but no evidence of any meridional reflections. X-ray scatter patterns from the cornea and hydrogel are shown on the same scale for comparison.

In accordance with our previously published results from 5 human corneas [4], small-angle X-ray scattering patterns from the centre of corneas C1–3 (with a water content of 72%, 76% and 80%) produced a series of meridional reflections arising from the regular D-period of native fibrillar collagen, which indexed on 65 nm (Fig. 6A). The three corneas (C1–3) also produced an equatorial reflection, caused by the uniformity of fibril diameters (measuring on average 33 ± 2 nm) and the regular Bragg spacing of collagen fibrils (51 ± 5 nm), within each lamella of the corneal stroma. The four lobes of increased equatorial scatter intensity result from the predominant orientation of corneal collagen in the superior-inferior and nasal-temporal directions.

The RHCIII hydrogels failed to produce a small-angle X-ray scatter pattern when examined in their naturally hydrated state (∼90% water content). However, negative staining with phosphotungstate ions (to enhance their collagen scatter), resulted in a decrease in their water content from 90% to 74% and the subsequent generation of X-ray scatter patterns showing diffuse equatorial scatter with two lobes of increased X-ray scatter intensity (Fig. 6B). Such patterns are characteristic of collagen fibres of varying diameter lying predominantly in the same direction. As clumped filaments act as a larger clustered, fractal-like component in terms of X-ray scattering they therefore contribute much more to the resulting scatter pattern than the fine filaments. Consistent with our previous TEM measurements of clumped filaments, the average diameter of the collagen fibres when measured by small-angle X-ray scattering was found to be 51.3 ± 1.7 nm. The X-ray scatter patterns generated from the biosynthetic construct showed no evidence of any meridional reflections even after negative staining.

## 4 Discussion

In this paper, we consider the relationship between the structural and functional properties of two proven biocompatible materials [22], namely the human cornea and RHCIII biosynthetic corneal hydrogels (with and without the incorporation of MPC). The structure of the hydrogel differs substantially from that of the human cornea, however, they share a common physical property that is essential to their function – transparency. In fact, it has been shown that both the cornea [1] and the RHCIII hydrogels (with and without MPC) transmit almost all the incident light in the visible spectrum (87% [36], 93%, and 90% [21] at 500 nm respectively). Since the majority of organic molecules do no absorb visible light [37], we need only consider the attenuation of light caused by scattering when attempting to explain the transparency of each material. In the absence of light absorption, the amount of light scattered by a tissue is dependent upon its thickness, the size and shape of its constituents and their refractive indices [38]. Since the 1960’s, most theories of corneal transparency have built upon the lattice theory proposed by Maurice [8], which stated that the scattering from individual fibrils could be estimated by approximating each fibril to a perfect, infinitely long cylinder. Due to the difference in refractive index between the narrow fibrils (of highly uniform diameter ∼33 nm) and their surrounding aqueous medium, a small amount of light is scattered from each fibril. However, the relatively ordered arrangement of fibrils in the cornea (which can be seen by electron microscopy), results in the destructive interference of light in all but the forward direction [8]. As the Beer–Lambert law states that light attenuation is exponentially proportional to path length, the thinness of the cornea is also key to its high level of transparency. In the same way, the transparency of the biosynthetic hydrogel may also be partially attributed to its thinness. However, we must also consider the size and shape of its constituents and their refractive indices. We have shown that the hydrogels have a very high water content (∼90%) and as seen by electron microscopy, contain long thin filaments (which are sometimes clumped into larger fibres) that are aligned parallel to the surface. The non-clumped fibres within the hydrogels are of a narrow diameter (with radii of 1–4.5 nm, far less than the wavelength of light) and the clumped fibres, which behave like larger particles in terms of light scatter, measure less than 80 nm in diameter and therefore also contribute very little. The final point to consider is the refractive index of the fibrils and the extrafibrillar material, and here it should here be noted that a completely homogenous refractive index is not always necessary for transparency – a tissue can have components of many different refractive indices so long as the average refractive index is constant over distances equal to half the wavelength of light or more [39]. Based on new and existing refractive index measurements, the total percent of incident light reflected at each interface between the epithelium and posterior stroma in the healthy cornea and RHCIII or RHCIII-MPC implanted corneas (discounting any wound healing effects) was predicted to be equally low at ∼2%. The transparency of the construct is therefore likely the result of its high water content, relatively sparse distribution of narrow collagen fibres and uniform refractive index.

Although a high level of transparency to visible light is obviously desirable for optimal vision, the transmission of UV light is potentially damaging to the lens and retina. The cornea contains a number of molecules that act as effective UV filters to protect the deeper structures of the eye from harmful wavelengths [40]. The corneal stroma absorbs approximately 70–75% of UV between 240 and 400 nm and the remainder is absorbed more or less equally by the epithelium and Bowman’s layer [35]. It is thought that the effectiveness of UV filtering in the stroma is due to its thickness (being approximately 10 times thicker than all other layers), whereas in the epithelium and Bowman’s layer it is due to their high absorption coefficients which are 2–7.5 times higher than that of the stroma at wavelengths below 300 nm [35]. In hydrogel-implanted corneas, stromal tissue containing cells and a range of extra-cellular matrix macromolecules, is replaced with a simple, single component highly UV transparent hydrogel, which is gradually repopulated by stromal cells and remodelled [22,24]. Although UV-C (which is potentially the most damaging UV radiation), is completely filtered out by the Earth’s atmosphere, and the most damaging short wavelength UV-B radiation is to a large extent absorbed by stratospheric ozone, UV-A passes through the atmosphere with little attenuation [41]. Patients should therefore be cautious about exposure to UV light, particularly in the period prior to epithelial regrowth (which occurs at approximately 1 month post-implantation, after removal of the sutures [22]) but also until the hydrogels are repopulated by stromal cells, which occurs within months [42] to several years, varying from patient to patient, [22,23]. Despite the obvious improvement in UV absorbance of the RHCIII hydrogels following the incorporation of MPC, our findings support the development of a new generation of hydrogels with UV filters built-in, or the use of protective sunglasses until corneal regeneration is complete.

In addition to being transparent, the cornea must also be strong enough to resist the forces exerted by both intraocular pressure and the extra-ocular muscles during eye movement, and thus maintain a precise curvature that is optimal for the focussing of light onto the retina. Although the incorporation of MPC into RHCIII hydrogels has led to enhancement in mechanical strength and a significant increase in stability when exposed to enzymes [24], the RHCIII-MPC hydrogels still remain considerably weaker than the cornea [21,24]. The difference in mechanical strength may be due to several factors, such as (1) the absence of proteoglycans from the hydrogels (which in the cornea help to maintain its structure), (2) the lack of collagen interweaving in the hydrogel versus the interwoven network of collagen in the anterior corneal stroma and the anchoring of fibrils into Bowman’s membrane (which result in the anterior-most 40% of the human cornea being considerably stronger than the rest of the tissue [43]), (3) the absence from the hydrogels of a plywood-like, stacked lamellar arrangement of collagen, which is present in the cornea and confers high tensile strength (Fig. 3), (4) the smaller diameter of collagen fibrils in the hydrogel (Fig. 3), and (5) the lower proportion of isotropic collagen (within the plane of the material) in the hydrogels (50%) compared to the cornea (70%). However, it should be noted when considering the results of strip extensometry that in the human cornea ∼30% of the total collagen mass arises from fibrils preferentially aligned in the nasal–temporal and superior–inferior directions (Fig. 5) [13–15] and at low strain rates (1%/min), strips cut from these ‘reinforced’ meridians display a greater tensile stiffness (resistance to deformation) than diagonally cut strips [44]. In the case of the hydrogels, ∼50% of the collagen is preferentially aligned in a uniaxial direction throughout (Fig. 5) (possibly due to a mechanism of self-alignment caused by molecular crowding during fibril assembly [45]. However, due to the ubiquitous external appearance of the hydrogels this ‘reinforced’ direction is unlikely to coincide with the direction of the cut strip, thus contributing to the low tensile strength of the hydrogels when tested using this method.

Clinical studies have shown that RHCIII implants are accepted into the host cornea and become epithelialized, innervated and populated with cells [22]. A decrease in the thickness of the hydrogel has been observed during the first 3 months post-operatively and a stabilisation thereafter [22]. The reduction in hydrogel thickness is presumably due to the negative charge of the proteoglycans in the surrounding corneal tissue drawing water out of the more highly hydrated hydrogel until an osmotic balance is achieved. Based on TEM studies of the hydrogels following standard processing (involving chemical dehydration) and high pressure freezing, such a decrease in hydration would be expected to lead to an increased incidence of filament clumping. As clinical studies have shown the implants to maintain a good level of transparency up to 24 months post-surgery, this supports our earlier statement that these clumped fibres contribute very little to light scatter [22]. Our X-ray scattering results have shown that at close to physiological corneal hydration the intermolecular spacing of collagen in the hydrogels is more or less equal to that of the cornea, but as shown by TEM, the diameter of the corneal collagen fibrils are much larger. This finding may be explained by the presence of a proteoglycan coating which surrounds each corneal collagen fibril and varies in diameter according to hydration [46] and also by the possibility that corneal collagen fibrils contain a larger number of collagen molecules per fibril than the hydrogels. At below physiological hydration, water is lost more readily from the hydrogel filaments than the corneal fibrils. This may be explained by the fact that when the cornea dries, water is first lost from between the fibrils and only when the tissue reaches a water content of below ∼50% is it also lost from the fibrils themselves [27,46], resulting in a decrease in collagen intermolecular spacing [27]. It appears that the hydrodynamic behaviour of the hydrogel is somewhat different to the cornea and water is much more readily lost from the hydrogel filaments; this is possibly due to their thinness or differences that may exist between the water binding capacity of the different collagen types that predominate in the cornea (type I) and hydrogel (type III).

The absence of collagen D-banding (such as seen in the cornea by both electron microscopy and X-ray scattering) indicates that “native” collagen fibrils are not formed within the biosynthetic hydrogels. This is not wholly surprising since in order to get reconstituted native-banded fibrils, the pH and ionic strength of the buffer is critical and even under optimal conditions in vitro reconstituted fibrils are not quite as well ordered as fibrils formed in vivo [47]. When comparing the structure and properties of the two materials it is however important to note that it may not be necessary for the hydrogel to be as strong as the cornea or to be comprised of native-banded fibrils onto which proteoglycans may be incorporated, if rather than existing as a functional component of the cornea the hydrogel is merely acting as a temporary scaffold to facilitate endogenous regeneration of the tissue. Clearly, further structural studies are needed to determine how remodelling occurs following implantation.

## 5 Conclusions

The structure of the biosynthetic hydrogels differs substantially from the human cornea but both have a high transparency to visible light. The transparency of the hydrogel may be attributed primarily to its high water content and narrow collagen filaments. Patients implanted with these hydrogels should exercise caution with regard to UV exposure, particularly in the period prior to epithelial regrowth.

## Disclosures

The authors have no conflicts of interest to disclose.

## Figures and Tables

**Table 1:** Predicted Fresnel light reflections at an incidence angle normal to the corneal surface in a normal cornea and an RHCIII-MPC implanted cornea (following epithelial regrowth).

Human cornea	Refractive index at each interface	Incident light reflected (%)	Human cornea with RHCIII-MPC hydrogel implant	Refractive index	Incident light reflected (%)
Air:tear film	1:1.33^a^	2	Air:tear film	1:1.33^a^	2
Tear film:epithelium	1.33^a^:1.401^b^	0.06	Tear film:epithelium	1.33^a^:1.401^b^	0.06
Epithelium:anterior stroma	1.401^b^:1.380^b^	0.006	Epithelium:hydrogel	1.401^b^:1.334	0.06
Anterior:posterior stroma	1.380^b^:1.373^b^	0.0006	Hydrogel:posterior stroma	1.334:1.373^b^	0.02

Published values for refractive index are cited from ^a^Craig et al. [31] and ^b^Patel et al. [32].
